# Acute compartment syndrome following Bitis viper envenomation: a literature review with case reports

**DOI:** 10.1186/s12245-025-01091-z

**Published:** 2025-12-22

**Authors:** Daša Baliarová, Kristian Chrz, Zdeněk Krška, Pavel Michálek, David Hoskovec, Jiří Valenta

**Affiliations:** 1 1st Surgical Department, General Teaching Hospital, U nemocnice 499/2, Prague 2, 128 08 Czech Republic; 2 1st Medical Faculty, Charles University, Kateřinská 1660/32, Prague 2, 128 08 Czech Republic; 3 Department of Anesthesiology and Intensive Care, General Teaching Hospital, Prague 2, 128 08 Czech Republic

**Keywords:** Compartment syndrome, Fasciotomy, Snakebite, Bitis nasicornis, Bitis gabonica

## Abstract

**Background:**

Snakebites caused by *Bitis nasicornis* and *Bitis gabonica* are rare but can lead to severe systemic and local complications, including acute compartment syndrome (ACS). The role of surgical intervention in snakebite management remains controversial, with limited data available for snakebite envenomation.

**Case presentation:**

Two cases of upper limb envenomation by Bitis nasicornis and Bitis gabonica were managed at the General University Hospital in Prague in year 2024. Both developed acute compartment syndrome requiring prompt antivenom therapy, fasciotomy, and intensive care. In the first case, antivenom (EchiTab-Plus-ICP) was given within 1 h, 10 vials in total, and fasciotomy at 10 h; the patient was discharged on day 16 with preserved limb function. In the second, antivenom (SAIMR) was administered within 3 h, 4 vials in total (the maximum available in Europe at that time), and fasciotomy at 8 h; recovery was complete by day 7. Diagnosis of ACS was based on clinical signs without intracompartmental pressure measurement.

**Conclusion:**

These cases highlight that timely surgical intervention, combined with intensive care and antivenin may play a critical role in preventing irreversible tissue damage following viperid envenomation. However, universal guidelines are lacking. Incorporating intracompartmental pressure monitoring into treatment protocols may further improve diagnostic accuracy and patient outcomes.

## Introduction

Venomous snakebites are relatively common problems worldwide. Relevant epidemiological data are virtually nonexistent because the obligation to report bites varies from country to country. According to the latest WHO data, an estimated **5.4 million snakebites** occur each year, resulting in **81**,**000–138**,**000 deaths** annually [1]. Deaths may occur as a direct consequence of venom toxicity (such as respiratory paralysis, coagulopathy, or renal failure) or as a result of secondary complications, including local tissue necrosis, infection, or delayed access to treatment. In the Czech Republic, there is no reporting obligation for bites by the common viper (Vipera berus), which naturally occurs at our latitudes, or for bites by exotic snakes. The Toxinology Centre (TC) recorded a total of 99 cases of breeders being bitten by exotic snakes between 1993 and 2018. In European countries, for example France [2], Germany [3] or Schwitzerland [4], the numbers are similar to those in the Czech Republic [5, 6]. An initial assessment of potential symptom development and the degree of envenomation risk requires at least a preliminary knowledge of the toxin composition of the venom and the typical venom yield of adult specimens. However, available data and practical experience remain limited, particularly for certain species within the genus Bitis—in this context, specifically *B. gabonica* and *B. nasicornis* [7].

### Bitis nasicornis and Bitis gabonica - brief characteristics, occurrence

**Fig. 1:**
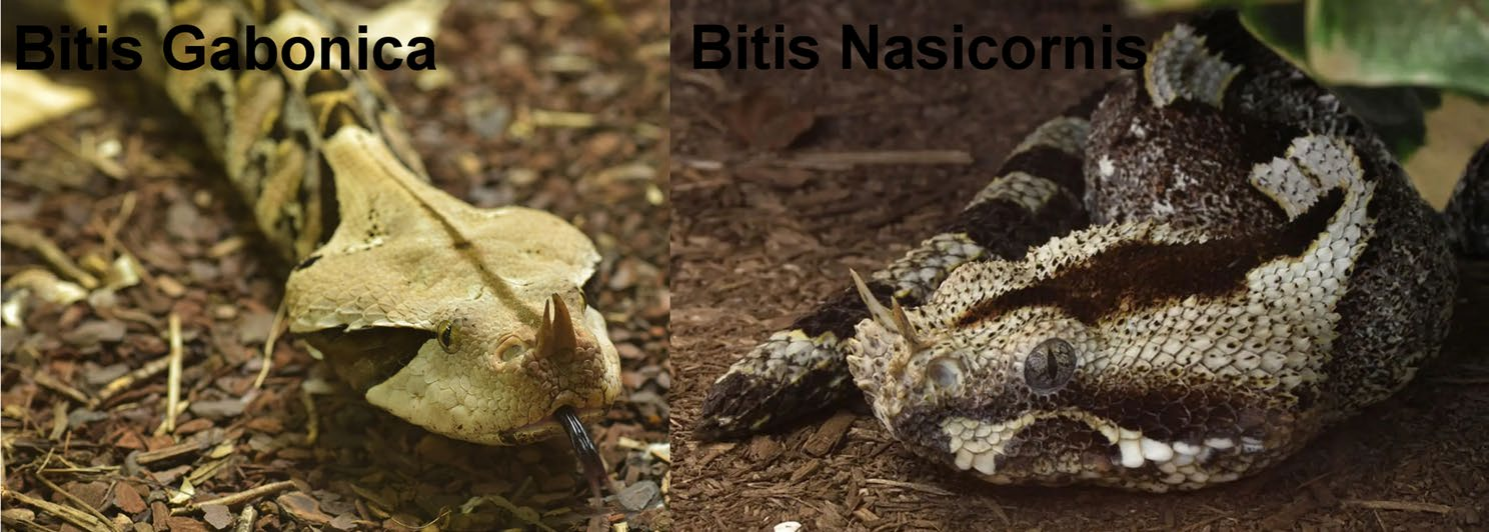
Image of Bitis gabonica (left) and Bitis nasicornis (right)

The genus *Bitis*, part of the Viperidae family [5, 8], comprises 17 species, broadly classified into ‘giant’ and ‘dwarf’ vipers [9, 10]. Our focus is on the giant species *B. gabonica* and *B. nasicornis (*Fig. 1*)*, both native to the rainforests of sub-Saharan Africa. These vipers are among the largest venomous snakes in Africa, often exceeding 1 m in length and capable of injecting large volumes of venom due to their robust size and extremely long fangs (up to 5 cm). Their presence in non-native regions such as Europe is almost exclusively linked to private keepers or breeders of exotic snakes [5, 6]. *B. nasicornis*, also known as the “butterfly viper” or “rhinoceros viper”, is a giant viper and one of the largest in Africa. They can grow to 100 cm in length but can be larger [10]. They are characterized by horn-like growth on the nose and are similar in color to Gabonese viper *B. gabonica* [10]. In areas where they are endemic, bites from these species are rarely reported. This may be due not only to their generally passive behavior and nocturnal habits but also to the fact that their natural habitats — dense rainforests — are often sparsely populated by humans, reducing the likelihood of human–snake encounters [11]. In sub-Saharan Africa, the attack viper (*B. arietans*) is reported to be responsible for the majority of snakebite deaths, with approximately 32 000 victims per year [12]. The focus of this communication is on *B. nasicornis* because, even in captivity outside Africa, only few case reports have been published, most of them from the U.S [8].

### Mechanism of action of snake venom of the family Viperidae

Venom from the Viper family (Viperidae) is a complex natural secretion primarily composed of biologically active proteins and enzymes, including phospholipase A2, metalloproteinases, serine proteinases, and phosphodiesterases, which contribute to its cytotoxic, myotoxic, hemotoxic, and procoagulant effects. While minor components such as carbohydrates, lipids, and amines may be present, the clinical impact is predominantly due to these enzymatic toxins [13–15]. Viper venom is also nephrotoxic, neurotoxic and especially hemotoxic, with disruption of coagulation and cardiovascular homeostasis, including impaired formation of stable thrombi, increased vascular permeability, and vasodilatation due to direct endothelial injury or indirect inflammatory mediators. Capillary leak syndrome may, in extreme cases, lead to so-called venom-induced consumption coagulopathy (VICC) [9, 16]. VICC is a type of venom-induced procoagulant coagulopathy that leads to pathological activation of the coagulation system by procoagulant toxins. The venom of certain viperid species — including *B. gabonica* and *B. nasicornis* — is characterized by excessive fibrinogen consumption and an increase in fibrinogen degradation products (FDPs) without effective fibrin clot formation. In practice, the best indicator and warning sign of incipient VICC is a prolongation of the PT and INR and increased D-dimer levels without or with the presence of hypofibrinogenemia [16–19].

These vipers are capable of injecting large amounts of venom—up to 1,000 mg per bite—thanks to their exceptionally long fangs, which may range from 2 to 5 cm depending on the individual. The intramuscular LD₅₀ of Bitis venom in experimental models is approximately 8.6 mg/kg [20], underscoring its potency. However, the actual volume of venom delivered in a bite varies significantly and depends on factors such as snake size or age, motivation, and prior depletion. The availability of antivenom is limited globally, including both endemic regions and high-income countries. In many African nations, access is further impeded by the **high cost**, limited distribution infrastructure, and **public mistrust**, often leading patients to seek traditional or alternative treatments. Furthermore, the **quality and efficacy of antivenoms vary significantly**, even when produced using venom from the same species. For example, two antivenoms derived from Bitis venom may differ markedly in neutralizing capacity and clinical outcomes [5, 16, 21, 22] These factors complicate management and emphasize the need for early, effective adjunctive treatments including surgical intervention in selected cases.

In 2016, the World Health Organization (WHO) revised the recommended management practices for the management of patients after snakebite and, among other things, clearly specified the indication criteria for the administration of antiserum. The clear indication criterion remains the development of general symptoms and/or the progression of severe local symptoms when adequate supportive therapy fails. Immunization against tetanus is part of the recommendation [16, 21].

### Clinical presentation after a bite by a snake of the family Viperidae

*Local symptoms* after viper bites include sharp pain, rapidly spreading swelling with erythema, lymphadenopathy, ecchymoses, bullae, and muscle necrosis with infectious complications [21, 22].

*Systemic manifestations* vary and may include gastrointestinal symptoms, hypotension to shock, tachyarrhythmia or bradyarrhythmia, and, only rarely, flaccid paralysi [18, 21–23].

*Compartment syndrome* following snakebite has a different pathophysiological basis than that following trauma. Venom-induced edema and pain can mimic compartment syndrome, and even after fasciotomy, the muscle is often not necrotic. Therefore, the diagnosis cannot rely solely on clinical presentation, and intracompartmental pressure measurement should be performed whenever feasible [24]. A pressure rise above 30 mm Hg should also raise suspicion of the suspected development of compartment syndrome [25, 26]. The incidence of compartment syndrome after snakebite is generally reported to be 4–15%, regardless of the species, so it can be said that it is a rare but serious complication [26, 27]. To date, there are almost no data on the incidence of compartment syndrome after Bites nasicornis and *B. gabonica* bites, probably because the number of documented cases worldwide is small. The mechanism of the development of acute compartment syndrome in a patient after a snakebite is different from that of trauma, and timely determination of the correct diagnosis and indication for surgical intervention may be difficult here because of the absence of recommended procedures.

The following two case reports illustrate the potential importance and benefit of timely surgical intervention in cases of snakebite-associated acute compartment syndrome and support the view that, in selected patients, surgery may have a meaningful role in preventing irreversible damage [28, 29].

## Case series

### Case 1

**Fig. 2:**
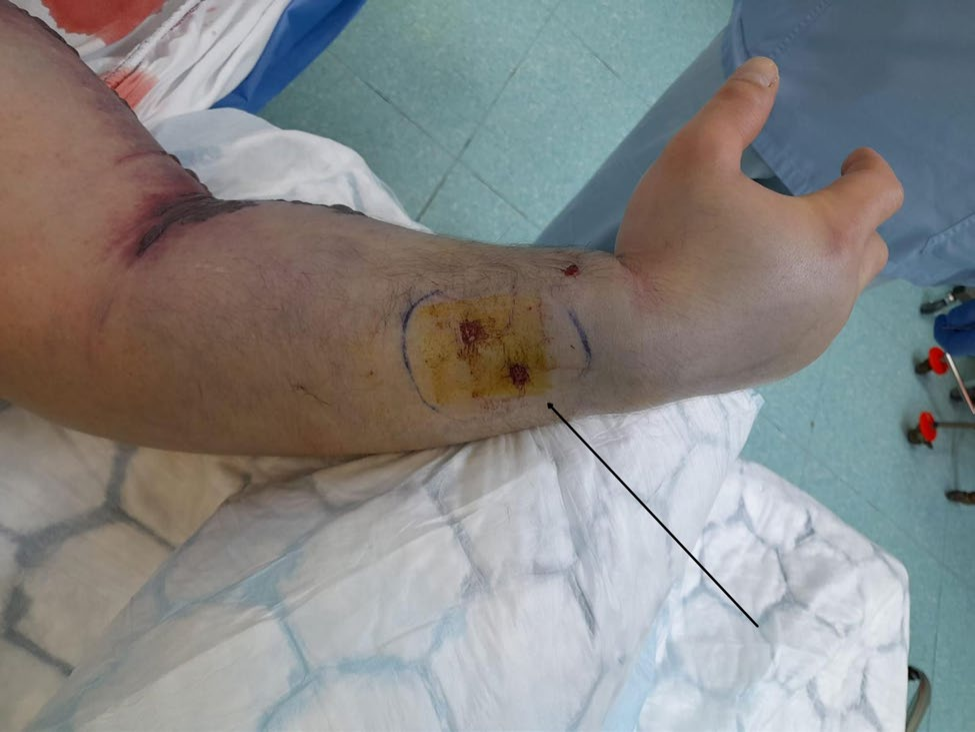
Limb with a visible snake tooth wound. The arrow indicates the bite site

A 40-year-old male, a domestic keeper of *B. nasicornis* (Fig. 1) with an unremarkable medical history, was brought to the Emergency Department of University Hospital in Pilsen on May 5 2024 at 4:50 p.m., following a bite to the dorsomedial right wrist by his snake at approximately 4:00 p.m. Initial symptoms included weakness, nausea, pain, swelling of the affected limb, and mild hypotension without respiratory compromise. By 5:00 p.m., he developed progressive shock requiring circulatory support and mechanical ventilation, coagulopathy, and hematoma formation (Fig. 2).

The first three doses (30 ml i.v.) of antivenom (EchiTab-Plus-ICP) were administered, and FAST ruled out internal bleeding, ATB prophylaxis started. He was rapidly transferred to the General University Hospital in Prague, arriving at 6:30 p.m.

**Table 1:** Laboratory parameters and vital signs of the patient in case 1 following *B. nasicornis* envenomation, including values at the time of initial antivenom administration, on arrival at the emergency department in Prague, and at the time of fasciotomy

Parameter	Pilsen University Hospital (antivenom start) 4:50 *p*.m.	ED arrival – Prague (antivenom continued) 6:30 *p*.m.	Fasciotomy start 02:00 a.m.
PT/INR	*Not available*	1.1	1.5
Fibrinogen (g/L)	*Not available*	1.5	2
Platelets (×10⁹/L)	*Not available*	248	161
Creatinin (µmol/L)	*Not available*	141	141
CK/Myoglobine (U/L, µg/L)	*Not available*	*Not available*/944	43.4/1192
Hemoglobine (g/L)	*Not available*	204	106
Lactate (mmol/L)	*Not available*	3.6	4.6
**Vital signs**			
Heart rate (HR)	120/min	75/min	109/min
Blood pressure (BP)	90/60 mmHg	110/60 mmHg	110/60 mmHg (0.5 ug/kg/min NOR)
Respiratory rate (RR)	16/min	18/min (UPV)	18/min (UPV SIMV 550 ml)
Oxygen saturation (SpO2)	*Not available*	96%	95%
Body temperature	36.8 °C	36.0 °C	38.5 °C

**Fig. 3:**
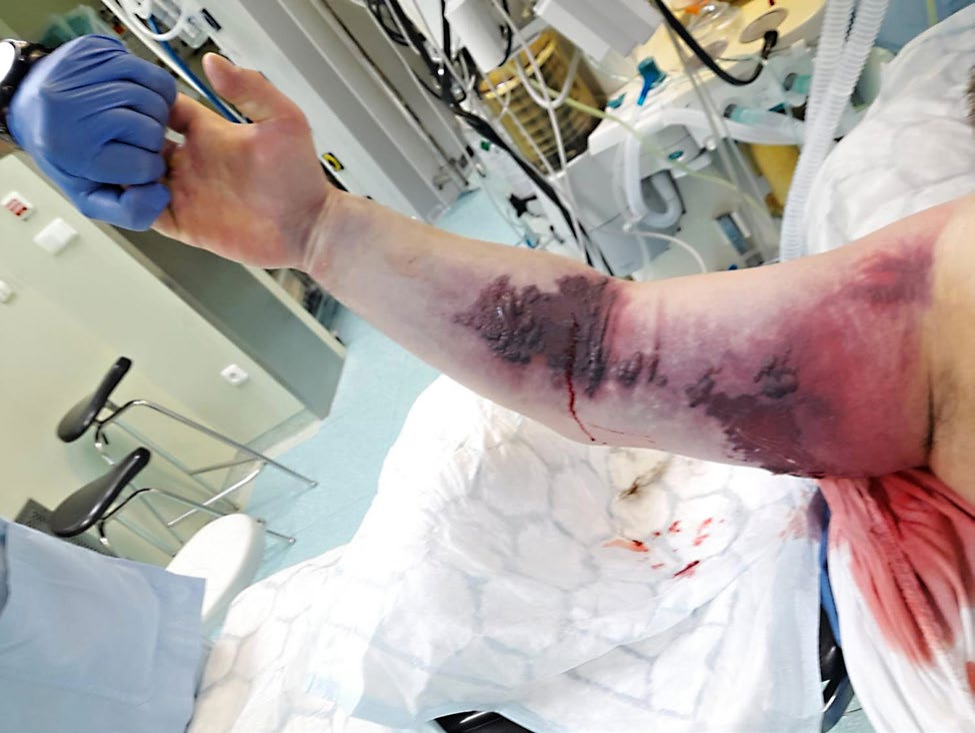
Limb shortly before fasciotomy. Approximately 7 h after injury, progression of right upper limb swelling with clinical manifestations of acute compartment syndrome - rest pain, paresthesias, ecchymoses

By 11:00 p.m., swelling had extended to the arm. The patient reported sharp resting pain aggravated by passive extension, paresthesia, and impaired mobility. Hemorrhagic bullae appeared on the inner arm and ventral forearm (Fig. 3). Another seven doses of the same antivenom were administered (70 ml i.v.) for worsening of the local findings, while systemic status remained stable.

**Fig. 4:**
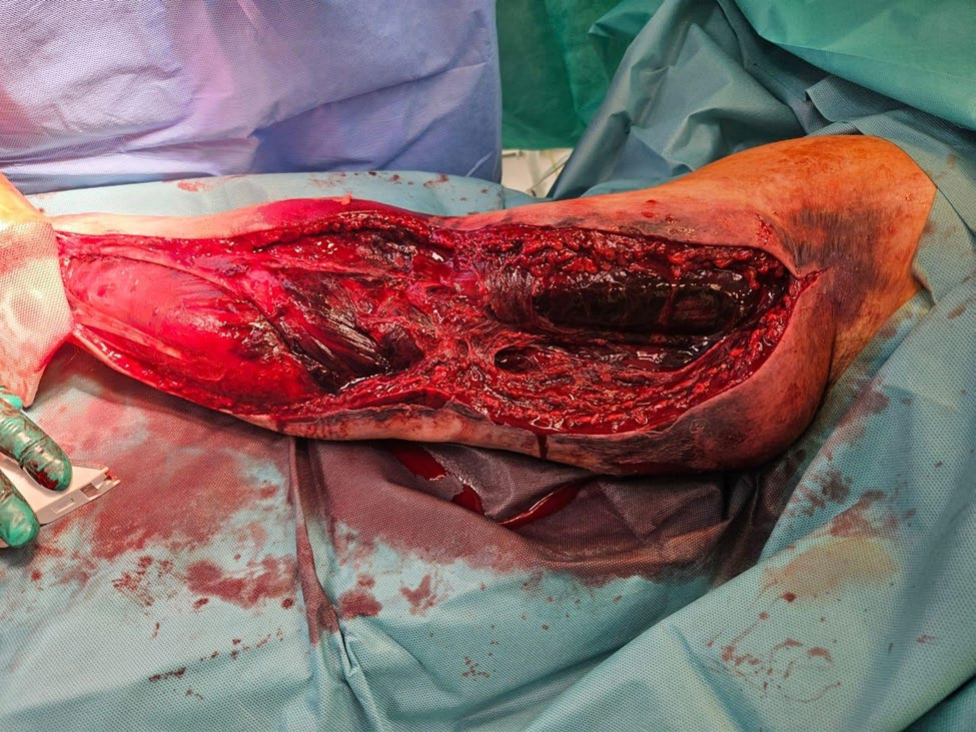
Limb recently subjected to fasciotomy

Intracompartmental pressure was not measured due to lack of equipment. The following table (Table 1) summarizes the patient’s laboratory. Fasciotomy was performed at 2:00 a.m. on May 6 based on clinical findings. A modified Henry’s approach, extended to the cubital and deltoideopectoral regions, was used. The transverse carpal ligament and lacertus fibrosus were cut. Muscles appeared viable but heavily hemorrhagic. (Fig. 4).

The following table (Table 2) summarizes the patient’s clinical course from day 2.

No adverse affects after the administration of the antiserum were observed.

**Table 2:** Clinical course of the patient in case 1 from postoperative day 2 onward, including hemodynamic status, surgical interventions, laboratory trends, and evolution of local limb findings following fasciotomy

Day	Clinical Status	Interventions / Findings	Limb Status
2	Progression of hemodynamic instability; septic shock	Adjustment of coagulation; slight renal impairment, persistent myoglobinemia, inflammatory markers slightly decreased	SpO₂ normal; limb periphery cold; no signs of compartment syndrome
3	Hemodynamics stabilized	Surgical revision and debridement; temporary defect coverage;	Limb warm; SpO₂ normal; no signs of compartment syndrome
4	Hemodynamically stable; reduced circulatory support	Surgical revision and debridement; temporary coverage; coagulation normalized; inflammatory markers low; extubation	Limb warm; SpO₂ normal; no signs of compartment syndrome
5–15	Stable; gradual de-escalation of circulatory support	Continuous antibiotics; tetanus immunization; CT scan ruled out bleeding; 4 additional surgical revisions (Days 4, 9, 10, 15) with debridement and temporary coverage	Limb mobility restored; satisfactory defect management
16	Discharge	Definitive coverage of defects planned by plastic surgeon; ATB terminated	Limb stable; ongoing recovery

### Case 2

A 55-year-old male patient, a private breeder of *Bitis gabonica*, was brought in by emergency medical services and transported by helicopter from Ústí nad Labem Hospital to the General University Hospital in Prague on July 7, 2024, at 4:30 p.m. The patient had reportedly been bitten on the right hand, in the area of the first dorsal interdigital space, at approximately 2:00 p.m. the same day. His medical history included dilated cardiomyopathy with an ejection fraction of 35%, chronic obstructive pulmonary disease (COPD), arterial hypertension, hyperlipoproteinemia, and obesity.

On admission, the patient presented with tachypnea, claudication, a tendency toward hypertension, swelling at the bite site (with two visible puncture wounds), impaired mobility, and capillary refill time < 2 s. Due to respiratory distress, intubation and mechanical ventilation were required. A CT scan of the trunk ruled out internal bleeding. ROTEM (Rotational thromboelastometry) revealed fibrinogen, plasma factor, and platelet deficiencies.

Antivenom (SAIMR Polyvalent Snake Antivenom) was administered on arrival, approximately two and a half hours after the bite (in total four vials 40 ml i.v. — the maximum available in Europe at that time). Additionally, 2 g of fibrinogen and 2000 IU of Prothromplex were administered, and antibiotic prophylaxis was initiated.

**Table 3:** Laboratory parameters and vital signs of the patient in case 2 following *B. gabonica* envenomation, including values at the referring hospital, on arrival at the general university hospital in Prague, and at the time of fasciotomy

Parameter	Ústí nad Labem Hospital 2:30 *p*.m.	ED arrival – Prague (antivenom start) 4:30 *p*.m.	Fasciotomy start 10:00 *p*.m.
PT/INR	1.5	1.6	1.6
Fibrinogen (g/L)	*Not available*	3	1.1
Platelets (×10⁹/L)	46	14	0
Creatinin (µmol/L)	*Not available*	73	80
CK/Myoglobine (U/L, µg/L)	*Not available*	1.37/21	2.8/212
Hemoglobine (g/L)	*Not available*	191	173
Lactate (mmol/L)	*Not available*	2.1	3
**Vital signs**			
Heart rate (HR)	100/min	125/min	105/min
Blood pressure (BP)	129/85 mmHg	140/80 mmHg	130/82 mmHg (0.3 ug/kg/min NOR + Vasopresin 3 IU/h)
Respiratory rate (RR)	20/min	NIV PS 6, PEEP 6	Spont PS 14, PEEP 8
Oxygen saturation (SpO2)	*Not available*	90%	95%
Body temperature	36.8 °C	36.8 °C	38.4 °C

Eight hours after the injury, swelling of the affected limb progressed, initially visible only on the hand but rapidly spreading to the forearm and arm. Capillary refill time was < 2 s, the limb was cool, and ecchymoses began to appear on the inner arm, the mobility worsened and rest pain in the limb appeared. Despite antivenom administration and maximal supportive care, the patient’s hemodynamic status deteriorated rapidly consistent with distributive shock. Urgent fasciotomy was indicated. Again, the Intracompartmental pressure was not measured due to the same reasons.

An incision was made from the linea vitae to the deltoideopectoral interval, the transversal carpal ligament was cut as well as the lacertus fibrosus, the median nerve was decompressed. The forearm muscles appeared viable, while the arm muscles were mildly hemorrhagic. The following table (Table 3) summarizes the patient’s laboratory.

By the second day of hospitalization, his hemodynamic status and renal parameters had stabilized, and ROTEM values normalized, the swelling did not progress, the mobility improved.

The following table (Table 4) summarizes the patient’s clinical course from day 3.

No adverse affects after the administration of the antiserum were observed.

**Table 4:** Clinical course of the patient in case 2 from postoperative day 3 onward, including hemodynamic stabilization, surgical revisions, respiratory status, and progression of local limb findings leading to discharge

Day	Clinical Status	Interventions / Findings	Limb Status
3	Persistent swelling; tissues viable	Surgical revision with debridement and temporary defect coverage; coagulation stabilized; inflammatory markers increased; renal function normal; tetanus immunization	Capillary refill < 2 s; SpO₂ normal; tissues vital; no signs of compartment syndrome
4–6	Hemodynamic stabilization; no vasopressors needed	Extubation; surgical revision with partial wound closure, debridement, temporary defect coverage; renal and coagulation parameters normal; inflammatory markers decreasing; Continuous ATB	Tissues vital; capillary refill normal; no signs of compartment syndrome
7	Clinically stable; ready for discharge	Termination of antibiotic prophylaxis	High likelihood of complete wound healing; no plastic surgery required

## Discussion

The care of patients after snakebite is a multidisciplinary collaboration in which, in addition to intensive care physicians and internists, nurses and rehabilitation workers, surgical care and intervention play a significant role. Because of the latency of symptoms, any patient bitten by a snake should be hospitalized for observation for at least 12 h, hospitalization should be extended to 24 h for patients who have received antiserum, and children should always be hospitalized [30].

Although acute compartment syndrome is a secondary complication in this case, without early surgical intervention, it can lead to loss of the affected limb or tissue necrosis with secondary infection or permanent impairment of mobility [31]. In trauma, acute compartment syndrome is clearly defined, and its clinical presentation is sufficient to indicate fasciotomy. It is not known how much time is required to develop irreversible tissue damage during ischemia. In general, the necessary duration of tissue ischemia leading to irreversible damage is reported to be at most 12 h [32]. The onset of typical local symptoms after snakebite is highly variable and ranges from 6 to 72 h [26]. The ideal time frame to administer an adequate dose of the appropriate antiserum is 4 h after the bite [33, 34]. However, in the case of snakebites, universal guidelines have not yet been established, i.e., when and under what circumstances to intervene surgically and whether it is a compartment syndrome at all, given that the mechanism of occurrence is different and depends on many other factors; however, these guidelines are unknown in most documented cases, and there is no major comprehensive study whose results could be considered significant and consistent in terms of the variables examined.

Newman et al. studied the incidence of snakebite-related compartment syndrome in the United States over the last 40 years and of 88 relevant and available records; only 12 such cases were reported, and only two of these had fasciotomies performed, which is still very low in relation to the average number of 6000 snakebites per year, even taking into account that the true incidence is somewhat higher. However, the results regarding laboratory values, intracompartmental pressure measurements or snake species in specific cases are very inconsistent [27].

Typical symptoms such as pain may overlap with the local effects of the bite itself, while absent pulses can result from venom-induced circulatory failure. It also depends on whether the bite is to the upper or lower limb. In the case of the lower limb, protective footwear and clothing often prevent deep penetration by the snake’s fangs. Therefore, unless the bite occurs through minimal or absent protection and the fangs reach the underlying muscle compartment, the risk of developing compartment syndrome is significantly reduced [35].

According to the most recent recommendations, a diagnosis of acute compartment syndrome should be considered only if circulatory failure has been successfully treated after the administration of antiserum or coagulation factors concomitant with supportive therapy, objective symptoms exist, and the intracompartmental pressure is greater than 40 mmHg (in adults). While intracompartmental pressure measurement has been described in the literature for over two decades, its incorporation into clinical decision-making protocols for **snakebite-induced** compartment syndrome has only more recently gained wider recognition and use in some centers [33, 34].

The results of Yon Hun Kim et al. revealed that even after the implementation of intracompartmental pressure measurement in 2014, fasciotomy was indicated in 17 out of 33 patients, i.e., 51.6%, whereas in 2010–2013, it was indicated in only 2 patients. This number is relatively high considering the overall reported worldwide incidence. The possible cause is suggested by the failure to adhere to the ideal time window for antiserum administration and that the majority of cases involved the upper limb (70.6%). It is not known which specific snake species were involved in each case. They, in turn, attributed the low number prior to the introduction of the measurements to a possible underdiagnosis, where pain and ischemic symptoms were thought to be effects of the venom [36].

Darracq et al., retrospectively for the period from January 2001–May 2012, identified 105 cases of patients following a Viperidae snakebite in whom fasciotomy was either performed or at least considered. Among these 105 patients, only 27% eventually underwent fasciotomy, and the intracompartmental pressure was measured in only 7% of them. All were given a specific antiserum, but it is not clear from the results at what time interval after the bite or whether these patients had circulatory failure at the time of diagnosis. Hospitalization was prolonged by two days in patients after fasciotomy. Again, the relevance and necessity of fasciotomy are questioned given that most patients are successfully treated conservatively, and the importance of intracompartmental pressure measurement as an objective method that can avoid unnecessary indications is emphasized [29]. This argument is supported by Cumpston et al. in a paper that has been cited extensively in relation to this topic. They analyzed 640 cases of snakebite from the subfamily Crotalinae of the viperid (Viperidae) family in animal models and concluded that modern antisera reduce intracompartmental pressure, increase tissue perfusion, and reduce hemorrhagic manifestations. Thus, he argues against surgical intervention and highlights the essential role of antisera because there are no studies to support the fact that fasciotomy reduces intracompartmental pressure or manifestations of myonecrosis [28].

Our work is limited by the number of patients, but both patients developed clinical signs of acute compartment syndrome within 8 h of being bitten on the upper limb by snakes with an average fang length of approximately 20 mm, so it is almost certain that the bite also caused injury to the musculature. Other work also confirms that anatomical location can be important in diagnostic reasoning. Yun Kim et al. reported that 12 of 17 (51.6%) fasciotomies at their institution were performed on the upper limb and that only 5 (29.4%) were performed on the lower limb [36].

In our work, we are limited by the number of patients; however, in both cases there was an objective development of compartment syndrome signs on the affected limb—practically within two hours after the bite (in both cases the right upper extremity). The condition continued to worsen over time despite maximized supportive therapy and administration of several vials of the appropriate antiserum. Both patients were hemodynamically stable at the time of fasciotomy.

In Case 1, following a *Bitis nasicornis* bite, the patient became hemodynamically unstable approximately one hour after the injury, requiring circulatory support and mechanical ventilation. He received the first three vials of antiserum within one hour after the bite. Upon arrival at the Emergency Department in Pilsen, typical signs of compartment syndrome were present—mobility impairment, a cold limb, rapidly progressive swelling, the gradual appearance of ecchymoses, and resting pain unresponsive to analgesics. Three hours after the bite, the patient received another seven vials of the appropriate antiserum. Although hemodynamic stability was achieved, the coagulation parameters (fibrinogen and platelet count) were getting worse, the local findings also continued to worsen; swelling extended from the hand to the arm, and hemorrhagic bullae appeared on the arm and forearm. Intracompartmental pressure was not measured due to the absence of the necessary equipment in our hospital. Eight hours after the bite, the surgeon indicated and performed fasciotomy. After the procedure, the patient again became hemodynamically unstable, but the local findings stopped progressing. By the third postoperative day, the patient was hemodynamically stable, had normalized coagulation parameters, and the local condition continued to improve until discharge.

In Case 2, following a *Bitis gabonica* bite, the patient remained hemodynamically stable for the first two hours after the injury. His condition, however, began to deteriorate rapidly upon arrival at our hospital, requiring circulatory and ventilatory support. Immediately after admission—two and a half hours after the injury—he received four vials of the appropriate antiserum (the maximum available in Europe at that time). Although hemodynamic stabilization and satisfactory coagulation parameters were achieved, the local findings continued to worsen. Swelling spread from the hand to the arm; the patient gradually developed mobility impairment, paresthesias, and resting pain, and ecchymoses appeared on the arm and forearm. Intracompartmental pressure was again not measured for the same reasons. After the surgical procedure and until discharge, the patient remained hemodynamically stable with normalized coagulation parameters.

In both cases, the bites were inflicted by vipers of the genus *Bitis*, which have an average fang length of 50 mm, and the bites occurred on the upper extremity—an area with a higher likelihood of compartment syndrome development, also because this part of the body is often not protected by clothing and direct muscle injury with injection of a high venom dose is presumed [33]. Our findings suggest that compartment syndrome cannot be managed solely with supportive therapy and timely administration of an adequate amount of antiserum, as in both cases the local condition continued to deteriorate despite these measures. Both patients developed objective signs of compartment syndrome—resting pain, progressive swelling, paresthesias, mobility impairment, hemorrhage—as well as the fact that after fasciotomy the progression stopped, the local condition improved, full mobility returned, and the pain syndrome and paresthesias resolved. Although intracompartmental pressure was not measured in either case, all other recent recommended criteria for fasciotomy were fulfilled [31, 32].

According to WHO snakebite guidance, antivenom should be administered as soon as there is clinical or laboratory evidence of systemic envenoming, or when local swelling is rapidly progressing and threatens to compromise tissue viability. Furthermore, the earlier antivenom is given after the onset of systemic or severe local envenoming, the more effective it is [19].

In both of our cases, the first vial of antivenom was administered within two hours of the injury and continued until hemodynamic stability was achieved consistent with the general consensus of multiple vials administration prior to fasciotomy [37]. Yet in neither case did this prevent the local tissue swelling and other signs consistent with compartment syndrome.

**Figure:**
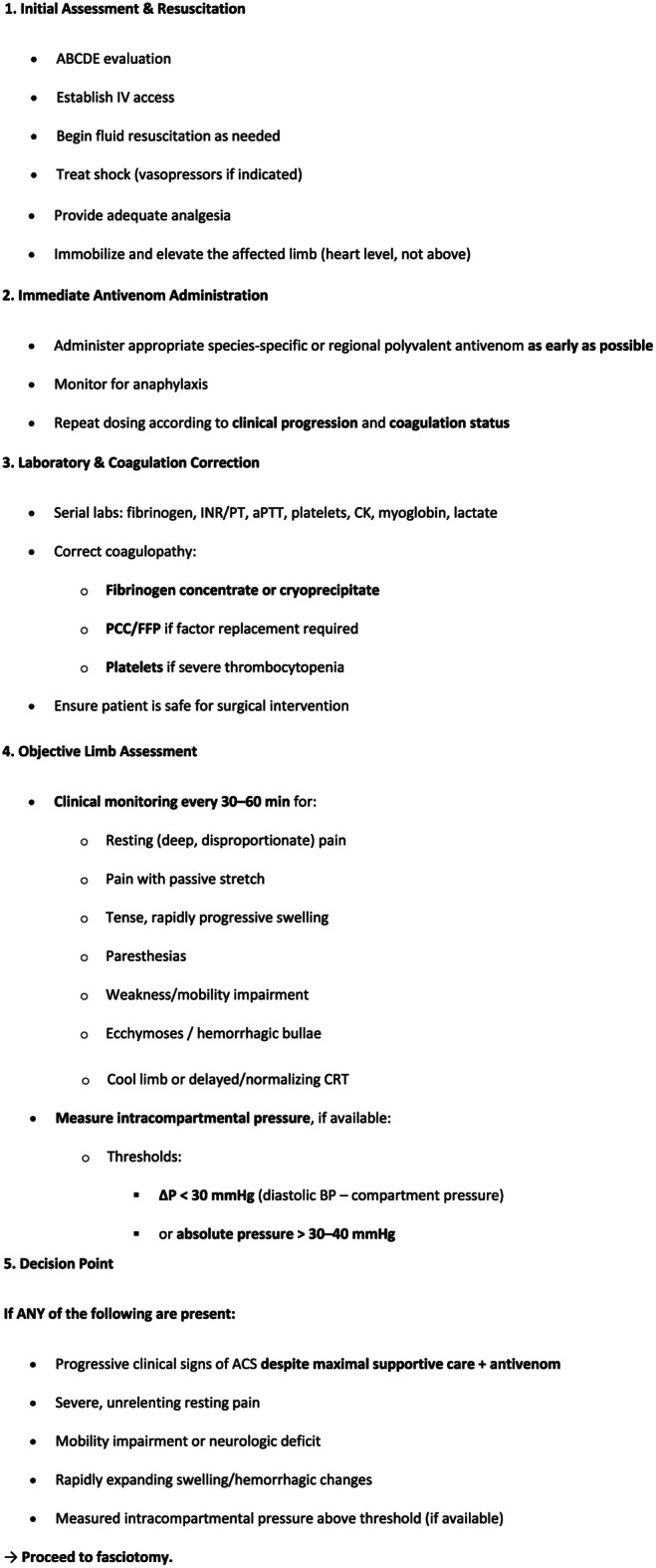
Algorithm for suspected acute compartment syndrome (ACS) after viperid snakebite

## Conclusion

Our paper is not intended to underestimate the importance of the specific immunotherapy and supportive therapy that was provided to our patients in the intensive care unit, but it certainly argues against claims that fasciotomy should be completely excluded from the treatment process [28].

Owing to the absence of a probe to measure intracompartmental pressure at our department, the indication for fasciotomy was only clinical. In our clinic, we encounter venomous snakebites relatively frequently compared with the rest of the country, and the implementation of new recommended procedures, especially the measurement of intracompartmental pressure, could lead to better outcomes for patients and could lead to the collection of data to determine the true incidence of compartment syndrome after snakebite.

## Data Availability

No datasets were generated or analysed during the current study.

## Abbreviations

UPV: Unassisted/Unconventional Pressure Ventilation
SIMV: Synchronized Intermittent Mandatory Ventilation
NOR: Noradrenaline
TC: Toxicologic Centre
NIV: Non – invasive ventilation
PS: Pressure support
PEEP: Positive End – Exspiratory Support
COPD: Chronic obstructive pulmonary disease
ROTEM: Rotational thromboelastometry

## References

[1] World Health Organization. Snakebite envenoming – Key facts. WHO Fact Sheet. September 2023. https://www.who.int/news-room/fact-sheets/detail/snakebite-envenoming

[2] De Haro L. Management of snakebites in France. Toxicon [Internet]. 2012 [cited 2025 Nov 14];60:712–8. 10.1016/j.toxicon.2012.03.013 10.1016/j.toxicon.2012.03.013 22465493

[3] Schaper A, Desel H, Ebbecke M, Haro LD, Deters M, Hentschel H, et al. Bites and Stings by exotic pets in europe: an 11 year analysis of 404 cases from Northeastern Germany and southeastern France. Clin Toxicol [Internet]. 2009;47:39–43. 10.1080/15563650801954875. [cited 2025 Nov 14];. 10.1080/15563650801954875 18608301

[4] Plate A, Kupferschmidt H, Schneemann M. CME: Giftschlangenbisse in der Schweiz. Praxis [Internet]. 2016 [cited 2025 Nov 14];105:679–85. 10.1024/1661-8157/a002388 10.1024/1661-8157/a002388 27269771

[5] Valenta J. Zmijí uštknutí? Těžké průběhy s úmrtími jsou spíše historickými skutečnostmi [Internet]. WwwCestomilacz - Portál O Cest. 2014. https://www.cestomila.cz/clanek/1220-jiri-valenta-zmiji-ustknuti-tezke-prubehy-s-umrtimi-jsou-spise-historickymi-skutecnostmi

[6] Valenta J, Stach Z, Michalek P. Exotic snake bites in the Czech Republic–Epidemiological and clinical aspects during 15-year period (1999–2013). Clin Toxicol Phila Pa. 2014;52:258–64. 10.3109/15563650.2014.902066. 10.3109/15563650.2014.902066 24666339

[7] Malina T, Krecsak L. Clinical aspects and consequences of envenoming by a captive rhinoceros Viper (Bitis nasicornis) in Hungary. Swiss Med Wkly [Internet]. 2008. 10.4414/smw.2008.12063. [cited 2024 Dec 6]. 18293116 10.4414/smw.2008.12063

[8] Moloo A. Snakebite envenoming: Member States provide WHO with clear mandate for global action [Internet]. 2018. https://www.who.int/news/item/25-05-2018-snakebite-envenoming-member-states-provide-who-with-clear-mandate-for-global-action

[9] Youngman NJ, Debono J, Dobson JS, Zdenek CN, Harris RJ, Brouw BOD et al. Venomous landmines: clinical implications of extreme coagulotoxic diversification and differential neutralization by antivenom of venoms within the Viperid snake genus Bitis. Toxins [Internet]. 2019 [cited 2024 Dec 6];11:422. 10.3390/toxins11070422 10.3390/toxins11070422 PMC6669450 31331004

[10] Spawls S, Branch B. The dangerous snakes of africa: natural history, species directory, venoms, and snakebite. Sanibel Island, FL: Ralph Curtis-Books; 1995.

[11] Boyer D. Notes on the natural history and husbandry of the rhinoceros Viper (Bitis nasicornis). Reptiles. 1195;1995:3:9–12.

[12] Olaniyan OV, Its Effect on Blood Chemistry of Envenomated Wistar Rats. Isolation and Purification of Bitis arietans (Puff Adder) Venom Proteins and. Annals of Psychiatry and Clinical Neuroscience [Internet]. 2022;5(1): 1045. https://www.remedypublications.com/open-access/isolation-and-purification-of-bitis-arietans-puff-adder-venom-proteins-9188.pdf

[13] Lu Q, Clemetson JM, Clemetson KJ. Snake venoms and hemostasis. J Thromb Haemost [Internet]. 2005 [cited 2024 Dec 6];3:1791–9. 10.1111/j.1538-7836.2005.01358.x 10.1111/j.1538-7836.2005.01358.x 16102046

[14] MacKay N, Ferguson JC, McNicol GP. Effects of the venom of the rhinoceros horned Viper *(Bitis nasicornis)* on blood coagulation, platelet aggregation, and fibrinolysis. J Clin Pathol [Internet]. 1970;23:789–96. 10.1136/jcp.23.9.789. [cited 2025 Nov 11];. 4251326 10.1136/jcp.23.9.789 PMC476899

[15] Marsh NA, Whaler BC. The Gaboon viper (Bitis gabonica): Its biology, venom components and toxinology. Toxicon [Internet]. 1984 [cited 2025 Nov 11];22:669–94. 10.1016/0041-0101(84)90152-1 10.1016/0041-0101(84)90152-1 6395443

[16] Erazo-Martínez V, Posso-Osorio I, Ruiz-Ordoñez I, Castro-Herrera F, Castaño-Valencia S, Delgado-Mora T et al. Viperidae snake envenomation from a highly complex hospital in Southwestern Colombia. Heliyon [Internet]. 2024 [cited 2024 Dec 6];10:e26768. 10.1016/j.heliyon.2024.e26768 10.1016/j.heliyon.2024.e26768 PMC10907710 38434332

[17] Isbister G. Snakebite Doesn’t Cause Disseminated Intravascular Coagulation: Coagulopathy and Thrombotic Microangiopathy in Snake Envenoming. Semin Thromb Hemost [Internet]. 2010 [cited 2024 Dec 6];36:444–51. 10.1055/s-0030-1254053 10.1055/s-0030-1254053 20614396

[18] Berling I, Isbister GK. Hematologic effects and complications of snake envenoming. Transfus Med Rev [Internet]. 2015 [cited 2024 Dec 6];29:82–9. 10.1016/j.tmrv.2014.09.005 10.1016/j.tmrv.2014.09.005 25556574

[19] Gutiérrez JM, Ownby CL. Skeletal muscle degeneration induced by venom phospholipases A2: insights into the mechanisms of local and systemic myotoxicity. Toxicon [Internet]. 2003;42:915–31. 10.1016/j.toxicon.2003.11.005. [cited 2024 Dec 6];. 15019491 10.1016/j.toxicon.2003.11.005

[20] Mallow D, Ludwig D, Nilson G. True Vipers: natural history and toxinology of old world Vipers. Original ed. Malabar, Fla: Krieger Pub. Co; 2003.

[21] Organization WH. Guidelines for the management of snakebites second edition. Geneva: World Health Organization; 2018.

[22] Guidelines for the. Prevention and clinical management of snakebite in Africa. Brazzaville: World Health Organization; 2010.

[23] Pungerčar J, Križaj I. Understanding the molecular mechanism underlying the presynaptic toxicity of secreted phospholipases A2. Toxicon [Internet]. 2007 [cited 2024 Dec 7];50:871–92. 10.1016/j.toxicon.2007.07.025 10.1016/j.toxicon.2007.07.025 17905401

[24] Meyer S, Hartmann F, Stein P, Lenherr R, Fuchs J, Spahn DR. Massive coagulopathy caused by the bite of a crotalus Basiliscus snake. Anaesth Cases [Internet]. 2017;5:60–5. 10.21466/ac.FCOABBC.2017. [cited 2025 Nov 11];.

[25] Prasarn ML, Ouellette EA. Acute Compartment Syndrome of the Upper Extremity: Am Acad Orthop Surg [Internet]. 2011 [cited 2024 Dec 7];19:49–58. 10.5435/00124635-201101000-00006 10.5435/00124635-201101000-00006 21205767

[26] Elliott KGB, Johnstone AJ. Diagnosing acute compartment syndrome. J Bone Joint Surg Br. 2003;85:625–32. 12892179

[27] Newman J, Therriault C, White MS, Nogee D, Carpenter JE. Compartment syndrome following snake envenomation in the united states: A scoping review of the clinical literature. West J Emerg Med [Internet]. 2024;25. 10.5811/WESTJEM.18401. [cited 2024 Dec 8];. 10.5811/westjem.18401 PMC11254155 39028252

[28] Cumpston KL. Is there a role for fasciotomy in *Crotalinae* envenomations in North america? Clin toxicol [Internet]. 2011 [cited 2024 Dec 8];49:351–65. 10.3109/15563650.2011.597032 10.3109/15563650.2011.597032 21740134

[29] Darracq MA, Cantrell FL, Klauk B, Thornton SL. A chance to cut is not always a chance to cure- fasciotomy in the treatment of rattlesnake envenomation: A retrospective poison center study. Toxicon [Internet]. 2015;101:23–6. 10.1016/j.toxicon.2015.04.014. [cited 2024 Dec 8];. 25935457 10.1016/j.toxicon.2015.04.014

[30] Jiří V. Jedovatí hadi. Galén; 2008.

[31] Evers LH, Bartscher T, Lange T, Mailander P. Adder bite: an uncommon cause of compartment syndrome in Northern hemisphere. Scand J Trauma Resusc Emerg Med [Internet]. 2010;18:50. 10.1186/1757-7241-18-50. [cited 2024 Dec 11];. 20854675 10.1186/1757-7241-18-50 PMC2949668

[32] Sheridan GW, Matsen FA. Fasciotomy in the treatment of the acute compartment syndrome. J Bone Joint Surg Am. 1976;58:112–5. 1249096

[33] Hall EL. Role of surgical intervention in the management of crotaline snake envenomation. Ann Emerg Med [Internet]. 2001;37:175–80. 10.1067/mem.2001.113373. [cited 2024 Dec 11];. 11174236 10.1067/mem.2001.113373

[34] Gold BS, Dart RC, Barish RA. Bites of venomous snakes. N Engl J Med [Internet]. 2002;347:347–56. 10.1056/NEJMra013477. [cited 2024 Dec 11];. 12151473 10.1056/NEJMra013477

[35] Nuchprayoon I, Pongpan C, Sripaiboonkij N. The role of prednisolone in reducing limb oedema in children bitten by green pit vipers: a randomized, controlled trial. Ann Trop Med Parasitol [Internet]. 2008;102:643–9. 10.1179/136485908X311786. [cited 2024 Dec 11];. 18817605 10.1179/136485908X311786

[36] Kim YH, Choi J, Kim J, Chung YK. Fasciotomy in compartment syndrome from snakebite. Arch Plast Surg [Internet]. 2019;46:69–74. 10.5999/aps.2018.00577. [cited 2024 Dec 11];. 30685944 10.5999/aps.2018.00577 PMC6369054

[37] Kanaan NC, Ray J, Stewart M, Russell KW, Fuller M, Bush SP, et al. Wilderness medical society practice guidelines for the treatment of pitviper envenomations in the united States and Canada. Wilderness Environ Med [Internet]. 2015;26:472–87. 10.1016/j.wem.2015.05.007. [cited 2025 Nov 14];. 26433731 10.1016/j.wem.2015.05.007

